# Comparative genomic analysis of clinical and environmental *Vibrio vulnificus* isolates revealed biotype 3 evolutionary relationships

**DOI:** 10.3389/fmicb.2014.00803

**Published:** 2015-01-15

**Authors:** Yael Koton, Michal Gordon, Vered Chalifa-Caspi, Naiel Bisharat

**Affiliations:** ^1^Department of Medicine D, Emek Medical CenterAfula, Israel; ^2^Bruce Rappaport Faculty of Medicine, Technion – Israel Institute of TechnologyHaifa, Israel; ^3^Bioinformatics Core Facility, National Institute for Biotechnology in the Negev, Ben-Gurion University of the NegevBeer-Sheva, Israel

**Keywords:** aquaculture, microbial genome, *Vibrio vulnificus*, whole genome shotgun sequences, evolution, core genome, accessory genome

## Abstract

In 1996 a common-source outbreak of severe soft tissue and bloodstream infections erupted among Israeli fish farmers and fish consumers due to changes in fish marketing policies. The causative pathogen was a new strain of *Vibrio vulnificus*, named biotype 3, which displayed a unique biochemical and genotypic profile. Initial observations suggested that the pathogen erupted as a result of genetic recombination between two distinct populations. We applied a whole genome shotgun sequencing approach using several *V. vulnificus* strains from Israel in order to study the pan genome of *V. vulnificus* and determine the phylogenetic relationship of biotype 3 with existing populations. The core genome of *V. vulnificus* based on 16 draft and complete genomes consisted of 3068 genes, representing between 59 and 78% of the whole genome of 16 strains. The accessory genome varied in size from 781 to 2044 kbp. Phylogenetic analysis based on whole, core, and accessory genomes displayed similar clustering patterns with two main clusters, clinical (C) and environmental (E), all biotype 3 strains formed a distinct group within the E cluster. Annotation of accessory genomic regions found in biotype 3 strains and absent from the core genome yielded 1732 genes, of which the vast majority encoded hypothetical proteins, phage-related proteins, and mobile element proteins. A total of 1916 proteins (including 713 hypothetical proteins) were present in all human pathogenic strains (both biotype 3 and non-biotype 3) and absent from the environmental strains. Clustering analysis of the non-hypothetical proteins revealed 148 protein clusters shared by all human pathogenic strains; these included transcriptional regulators, arylsulfatases, methyl-accepting chemotaxis proteins, acetyltransferases, GGDEF family proteins, transposases, type IV secretory system (T4SS) proteins, and integrases. Our study showed that *V. vulnificus* biotype 3 evolved from environmental populations and formed a genetically distinct group within the E-cluster. The unique epidemiological circumstances facilitated disease outbreak and brought this genotype to the attention of the scientific community.

## Introduction

*Vibrio vulnificus*, like other potentially pathogenic halophilic vibrios, is part of the marine microbiota. It occurs in high numbers in molluscan shellfish and in temperate zones, and especially during the warmer months it reaches sufficient concentrations to cause clinical disease in human (Oliver, 1989). The bacterium is capable of causing primary septicemia following its ingestion, and secondary septicemia through skin lesions in individuals with underlying chronic diseases. People who are most susceptible to *V. vulnificus* infection usually suffer from a chronic liver disease, primarily cirrhosis or alcoholic liver disease, diabetes mellitus, or diseases associated with iron overload such as hemochromatosis and thalassemia major (Oliver, 2006). Worldwide, the vast majority of human disease has been reported from USA and Southeast Asia due to dietary habits of eating raw or undercooked seafood (Tacket et al., 1984; Klontz et al., 1988; Park et al., 1991; Chuang et al., 1992; Kumamoto and Vukich, 1998; Chiang and Chuang, 2003; Matsumoto et al., 2010). Reports from other parts of the world have been largely sporadic and typically due to wound infection (Bock et al., 1994; Melhus et al., 1995; Dalsgaard et al., 1996; Horre et al., 1998; Torres et al., 2002; Frank et al., 2006).

*V. vulnificus* populations have been divided into subpopulations based on phenotypic (biochemical) and genotypic characteristics. Phenotypically, three biotypes have been described; biotype 1, the most common worldwide (Oliver, 1989), biotype 2 mainly affecting eels (Tison et al., 1982), and biotype 3 that has been reported only in Israel (Bisharat et al., 1999). Several genotypic methods showed that *V. vulnificus* populations resolve into two main clusters, one dominated by strains from environmental sources and shellfish, and the other dominated by strains from human clinical samples (Nilsson et al., 2003; Bisharat et al., 2005; Gonzalez-Escalona et al., 2007). More recently a simple PCR-based assay showed excellent differentiation of *V. vulnificus* into two main genotypes based on their source of isolation, E-genotype (environmental) and C-genotype (clinical) (Rosche et al., 2005). Analysis of whole genome shotgun (WGS) sequence data confirmed this distinction and identified key genes specifically associated with each genotype (Morrison et al., 2012).

The disease outbreak in Israel in 1996 had several unique features. First, it was the first ever reported common source outbreak of *V. vulnificus* occurring among fish farmers and fish consumers handling live tilapia fish cultivated in inland fish farms (Bisharat and Raz, 1996). Second, biochemically the pathogen differed from existing pathogens, and was subsequently named biotype 3 (Bisharat et al., 1999). Finally, genotypically the bacterium displayed a unique pattern later identified as a hybrid clone of existing populations (Bisharat et al., 2005). Furthermore, because all disease cases were caused exclusively by a highly clonal pathogen (Bisharat et al., 1999, 2007a; Colodner et al., 2002, 2004; Miron et al., 2003; Zaidenstein et al., 2008), it was suggested that a recent genetic event have enabled a harmless environmental population to acquire capabilities to cause a deadly disease in human.

Our previous observations highlighted the importance of recombination in generating genetic diversity within *V. vulnificus* and showed that hybrids may be a recurring feature of *V. vulnificus* evolutionary biology (Bisharat et al., 2005, 2007b; Bishrat, 2010). However, the genetic divergence of *V. vulnificus* populations into two distinct genetic clusters was maintained across the genome and systematic over both chromosomes. Against a background of so much potential recombination we suggested two possible scenarios for the evolution of *V. vulnificus* populations, are the strains falling into two populations because of an old lineage split into two clonal complexes, which have accumulated differences through both mutation and horizontal transfer of diversity from a range of unknown sources, perhaps from other *Vibrio* species? Alternatively, is *V. vulnificus* a highly recombining species that structures into two populations for contemporary rather than phylogenetic reasons, implying some unknown ecological barrier that limits recombination between populations compared to within? (Bisharat et al., 2007b). Nevertheless, some inter-cluster hybrids do emerge and persist; the emergence of *V. vulnificus* biotype 3 was considered as such (Bisharat et al., 2005), yet we could not identify the parental lineage from which it had emerged. Recent observations based on WGS sequencing of a single biotype 3 strain implied that a single episode of genome hybridization of two bacterial populations is less likely to be the main event for the emergence of *V. vulnificus* biotype 3 and that it may have evolved by lateral gene transfer from other bacteria, such as *Shewanella* (Efimov et al., 2013). In order to elucidate the evolutionary pathways that led to the emergence of *V. vulnificus* biotype 3, we carried out a comparative genomic approach using three human pathogenic biotype 3 strains and two environmental strains, all isolated from Israel. We aimed to study the pan genome structure of *V. vulnificus*, identify the set of novel sequences characterizing the human pathogenic biotype 3 and determine its phylogenetic relationship with other human pathogenic and environmental populations.

## Methods

### Bacterial strains

We used five *V. vulnificus* strains for the study (Table 1): three biotype 3 strains isolated from Israeli patients with invasive infection—VV9-09, VV 4-03, and 491771—and two biotype 1 strains, 101/4 and 2322, isolated from fish and fish pond water in Israel, respectively. All biotype 3 strains belong to sequence type 8 as determined by multi-locus sequence typing (MLST) (Bisharat et al., 2005).

**Table 1:** **Characteristics of strains used in the study**.

**Strain**	**Biotype**	**Source**	**Year**	**Country**	**MLST^a^**
101/04	1	Fish	1997	Israel	59
2322	1	Water	1997	Israel	10
491771	3	Human	1997	Israel	8
VV9-09	3	Human	1999	Israel	8
VV 4-03	3	Human	2003	Israel	8

a *Sequence type as determined by multi-locus sequence typing (MLST)*.

### Genome amplification, building an illumina library, and sequencing

Genomic DNA was extracted using a commercial kit (Qiagen DNeasy kit). Adaptors were added to each library during preparation according to the TruSeq protocol (Illumina) to produce multiplexed paired-end libraries. Pools of four samples were run on a sequencer (Illumina MiSeq) at the Technion Genome Center, Haifa, Israel, generating 250 base paired-end reads.

### Read mapping, genome assembly, and annotation

Sequence read data were analyzed using a pipeline of initial analysis that consisted of mapping reads to reference genomes and variant calling, genome assembly and gene annotation. The reads were mapped to two reference genomes, CMCP6 and YJ016 (biotype 1 human pathogenic *V. vulnificus* strains with complete genome sequence). Reads were mapped to the reference genomes using Burrows-Wheeler Aligner (BWA) 0.6.1 (Li and Durbin, 2009). Raw sequence data were tested for quality control using FastQC (Andrews, 2014). Due to decreased quality, the last 25 bp in read2 were trimmed from all the samples, improving the mapping of these reads against the reference genomes with only a minor reduction in the average coverage (~5%). The trimmed reads were used for the next steps of the analysis. In order to determine how many mismatches to allow per read during mapping to reference genomes, several mapping attempts were made for each sample against the two reference genomes (5, 15, 20, 25 mismatches per read). Allowing 20 mismatches per read increased the percentage of unique mapping to the reference genomes, while 25 mismatches resulted in only a minor addition of unique mapping. We used Artemis (Rutherford et al., 2000) to visualize alignment of reads to the reference genomes and quantify the number of reads per gene in each sample. Reads per kilobase of exon model per million mapped reads (RPKM) values were calculated for each gene and ORFs in each sample. Identification of genes shared by the human pathogenic strains and absent from the environmental strains was estimated based on read coverage to the human-pathogenic and the environmental strains. Genes were regarded as “present” in a sample if they had more than 30 aligned reads, and “absent” if they had less than 30 such reads. CLC bio's de novo assembler (v 6.5) (Qiagen) was used for de novo assembly of all the samples, using default parameters. RAST (Rapid Annotation using Subsystem Technology) (Aziz et al., 2008) was used for gene annotation.

### Comparative genomics and phylogenetic analysis

The assembled genomes generated in the current study were used for comparative genome sequencing analysis together with three reference human-pathogenic strains with complete genome sequence; CMCP6 (RefSeq: NC_004459 and RefSeq: NC_004460), YJ016 (RefSeq: NC_005128; RefSeq: NC_005139; RefSeq: NC_005140), M06-24/O (RefSeq: NC_014965; RefSeq: NC_014966). Comparisons were also made with sequencing data from WGS sequencing projects available at http://www.ncbi.nlm.nih.gov/Traces/wgs/?, this included VVyb1 (biotype 3 strain isolated from tilapia fish in Israel) (NZ_AOCM00000000.1), BAA87 (biotype 3 strain isolated from human wound in Israel) (JDSE00000000.1), ATCC 27562 (biotype 1 strain isolated from human blood in the USA) (AMQV00000000.1), B2 (*V. vulnificus* strain isolated from human blood in China) (NZ_AMQR00000000.1). There were also three environmental strains isolated from oysters in the USA: JY1305 (AFSW00000000.1), E64MW (AFSX00000000.1), and JY1701 (AFSY00000000.1). In addition, we used WGS sequence data of a biotype 2 strain (ATCC 33147) isolated from an infected eel in Japan 1979, the draft genome of this strain was recently published by our group (Koton et al., 2014) (JRQR01000000). The pan-genome was studied using Panseq (Laing et al., 2010) with application of two main modules; the Core and Accessory Genome Finder (CAGF) and the Novel Region Finder (NRF). For the purposes of the analysis, the CAGF module considers the “pan genome” to be comprised of all sequences selected as input for the analysis. The software uses a sequence file as a seed to which all other sequences are compared using MUMmer (Delcher et al., 1999). If a segment greater than the “minimum sequence size” is found in other than the seed, that segment is added to the pan genome. Next, the software fragments the entire pan-genome into segments of user-defined length (in the current study we used the default measures of 500 bp), and determines the presence or absence of each of these fragments in each of the original sequences based on the percent sequence identity cutoff using the Basic Local Alignment Search Tool (BLAST) algorithm. Fragments above the cutoff (we used a percent nucleotide sequence identity cutoff ≥90%) found in every original sequence are considered part of the “core” genome, while fragments below the cutoff in at least one strain are considered part of the “accessory” genome. For the CAGF module, we used the assembled genomes of five biotype 3 strains: VV9-09, VV 4-03, 491771, VVyb1, and BAA87. The NRF module compares an input sequence(s) with a database of sequences and produces an output file of sequences found in the query sequences and absent from the reference strains.

REALPHY (reference sequence alignment based phylogeny builder) (Bertels et al., 2014) was used for the phylogenetic analysis using sequence data from the present study, and sequence data from WGS sequencing projects of eight *V. vulnificus* strains (VVyb1, BAA87, ATCC27562, B2, JY1305, E64MW, JY1701, and ATCC 33147). In addition, we used the complete genome sequence of three reference genomes (CMCP6, YJ016, and MO6-24/O). REALPHY infers phylogenetic trees from whole genome sequence data where all provided sequences are mapped to each of the references via bowtie2 (Langmead et al., 2009). From these alignments, phylogenetic trees are inferred via PhyML (phylogenetic estimation using maximum likelihood) (Guindon et al., 2010). The phylogenetic analysis was carried out using the draft whole genomes and then repeated using core and accessory genomes separately. The core and accessory genomes for this analysis were extracted using SPINE and AGEent (Ozer et al., 2014). SPINE identifies a core genome from genomic regions found among all submitted genomes (using default parameters; 100% of all input genomes in which sequence must be present to be considered “core,” and ≥85% identity of nucleotide alignments to be considered homologous). CD-HIT, a program for clustering large datasets of nucleotide or amino acid sequence data, was used for clustering protein sequences sharing sequence similarity among all the human-pathogenic strains and absent from all the environmental strains, using default parameters (amino acid sequence similarity ≥90) (Li and Godzik, 2006; Fu et al., 2012). The clustering algorithm is an incremental clustering algorithm. Briefly, sequences are first sorted in order of decreasing length. The longest sequence becomes the representative of the first cluster. Then, each remaining sequence is compared with the representatives of existing clusters. If the similarity with any representative is above a given threshold (amino acid sequence similarity ≥90), it is grouped into that cluster. Otherwise, a new cluster is defined with that sequence as the representative.

## Results

### Sequencing quality and read mapping to reference genomes

The quality of base calling from images and sequences was determined by quality score (*Q*). Approximately (average of 2 runs) 80% of inserts (paired end segments that were sequenced) passed the quality filter (*Q* = 30), indicating a 99.9% accuracy of base calling at a particular sequence position. Mapping statistics of all the strains to the reference genomes CMCP6 and YJ016 showed, as rather expected, that the human pathogenic strains showed higher rates of uniquely mapped reads to the reference genomes than the environmental strains.

Read mapping of three human-pathogenic biotype 3 strains (VV9-09, VV 4-03, and 491771) and two environmental biotype 1 strains (101/4 and 2322) to the reference genomes CMCP6 and YJ016 showed that between 64 and 66% of the reads obtained from biotype 3 strains were uniquely mapped (passed quality filter with up to five mismatches), while for the environmental strains only 55–58% of the reads were uniquely mapped. Based on the uniquely mapped reads we extracted the genes that were common to the human-pathogenic strains and absent from the environmental strains (File S1). Altogether there were 176 genes common to the human pathogenic strains, 71 genes (40.3%) encoded hypothetical proteins, while other major groups included genes encoding outer membrane assembly and transcriptional regulators. We identified 43,021 and 47,468 SNPs present in the human pathogenic strains and absent from the environmental strains which were also found in the reference genomes CMCP6 and YJ016, respectively.

The five Israeli samples—three human pathogenic biotype 3 strains and two environmental biotype 1 strains—were subjected to QUAST (Gurevich et al., 2013), a quality assessment tool for genome assemblies, using CMCP6 as a reference genome. We used the assembled genomes of two other biotype 3 strains, BAA87 and VVyb1, which were published by others for comparison purposes (Danin-Poleg et al., 2013; Phillips et al., 2014). The assembled genomes of biotype 3 strains (VV9-09, 491771, VV 4-03, BAA87, and VVyb1) showed similar characteristics and were different from the environmental strains (2322 and 101/04) (Table 2). This WGS project has been deposited at DDBJ/EMBL/GenBank under the accession IDs: JQDW00000000 (VV9-09), JQDV00000000 (VV4-03), JQDU00000000 (491771), JQDT00000000 (101/4), and JQDS00000000 (2322). The versions described in this paper are versions JQDW01000000, JQDV01000000, JQDU01000000, JQDT01000000, and JQDS01000000.

**Table 2:** **Quality assessment of assembled genomes**.

**Strain**	**101/04**	**2322**	**VV9-09**	**491771**	**VV 4-03**	**BAA87^a^**	**VVyb1^a^**
No. of reads	9,786,036	9,150,870	7,465,222	7,291,670	7,944,338	NA	NA
Average length – contigs (bp)	14,853	12,811	13,463	9,774	11,454	23,531	41,029
No. contigs (≥0 bp)	370	413	391	543	460	218	140
No. contigs (≥1000 bp)	63	78	177	200	190	187	115
GC (%)	46.32	46.4	46.42	46.43	46.42	46.49	46.73
N50	506,351	324,391	54,238	57,095	57,166	52,210	230,903
N75	143,535	161,992	33,564	34,718	33,212	32,756	109,426
No. of fully unaligned contigs	133	162	101	175	139	68	43
Fully unaligned length (bp)	170,338	152,003	326,751	390,311	369,492	284,904	226,696
No. mismatches per 100 kbp^b^	3717.52	3753.33	3024.61	3013.1	3022.73	3019.71	2875.88
No. indels per 100 kbp^c^	73.28	93.49	77.05	80.98	77.7	80.33	59.75
Genome fraction (%)^d^	63.6	61.4	79.8	78.9	79.4	79.9	79.3
No. predicted genes (≥0 bp)	5017	4836	4961	5068	5026	4907	5291
No. predicted genes (≥300 bp)	4380	4255	4206	4264	4196	4279	4699
No. predicted genes (≥1500 bp)	752	722	719	713	714	680	806
No. predicted genes (≥3000 bp)	85	84	73	71	72	63	78

*N50 is the length for which the collection of all contigs of that length or longer covers at least half an assembly. N75, defined similarly to N50, with 75% instead of 50%*.

a *Strains sequenced by others (Danin-Poleg et al., 2013; Phillips et al., 2014)*.

b *The average number of mismatches per 100,000 aligned bases. True SNPs and sequencing errors are not distinguished and are counted equally*.

c *The average number of indels per 100,000 aligned bases. Several consecutive single nucleotide indels are counted as one indel*.

d *Percentage of aligned bases in the reference*.

### Core and accessory genomes

**Figure 1:**
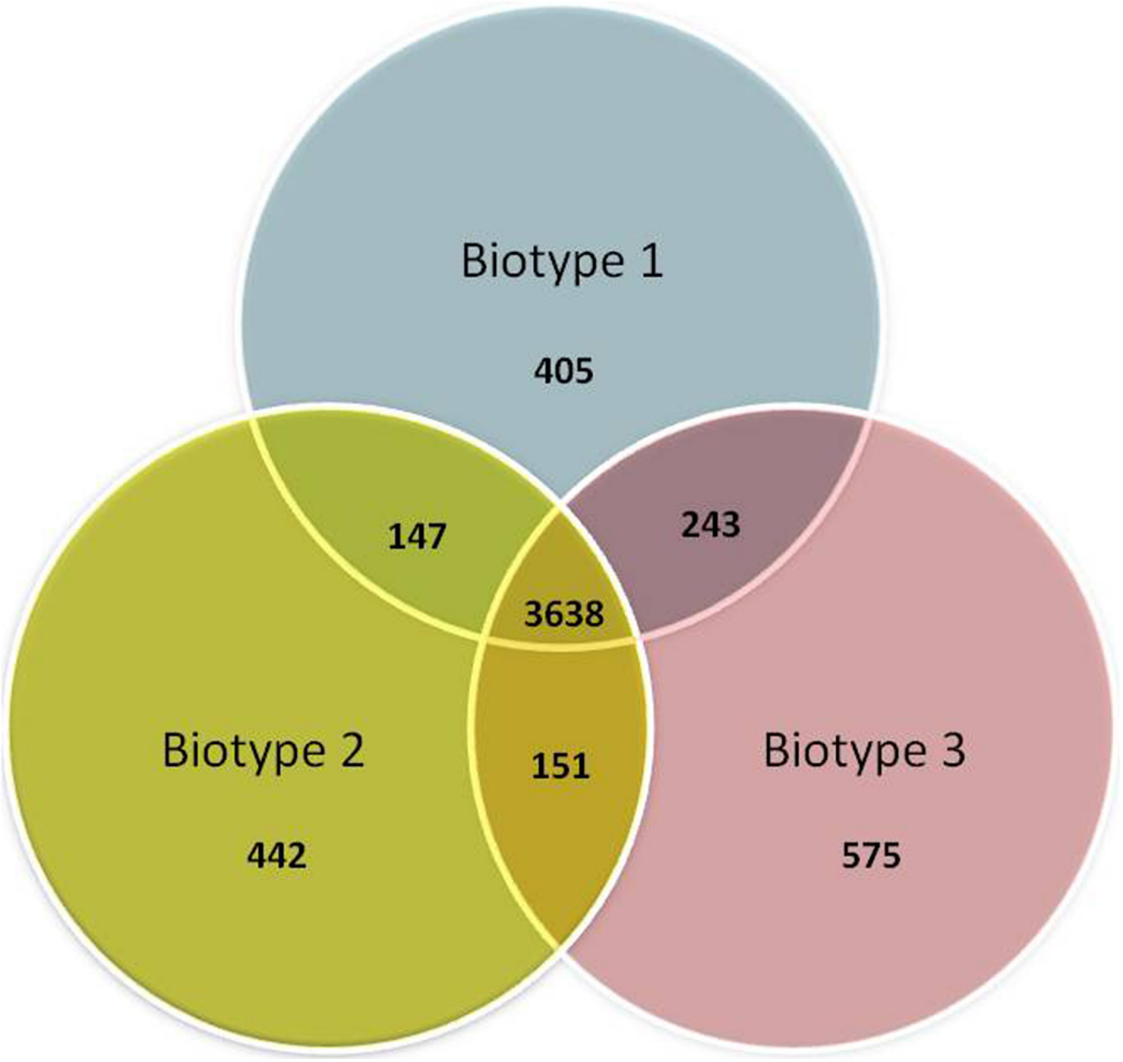
**Venn diagram representing differential and shared gene counts between representative strains of the three biotypes**. Biotype 1 = strain CMCP6, biotype 2 = strain ATCC 33147, and biotype 3 = strain 491771.

**Figure 2:**
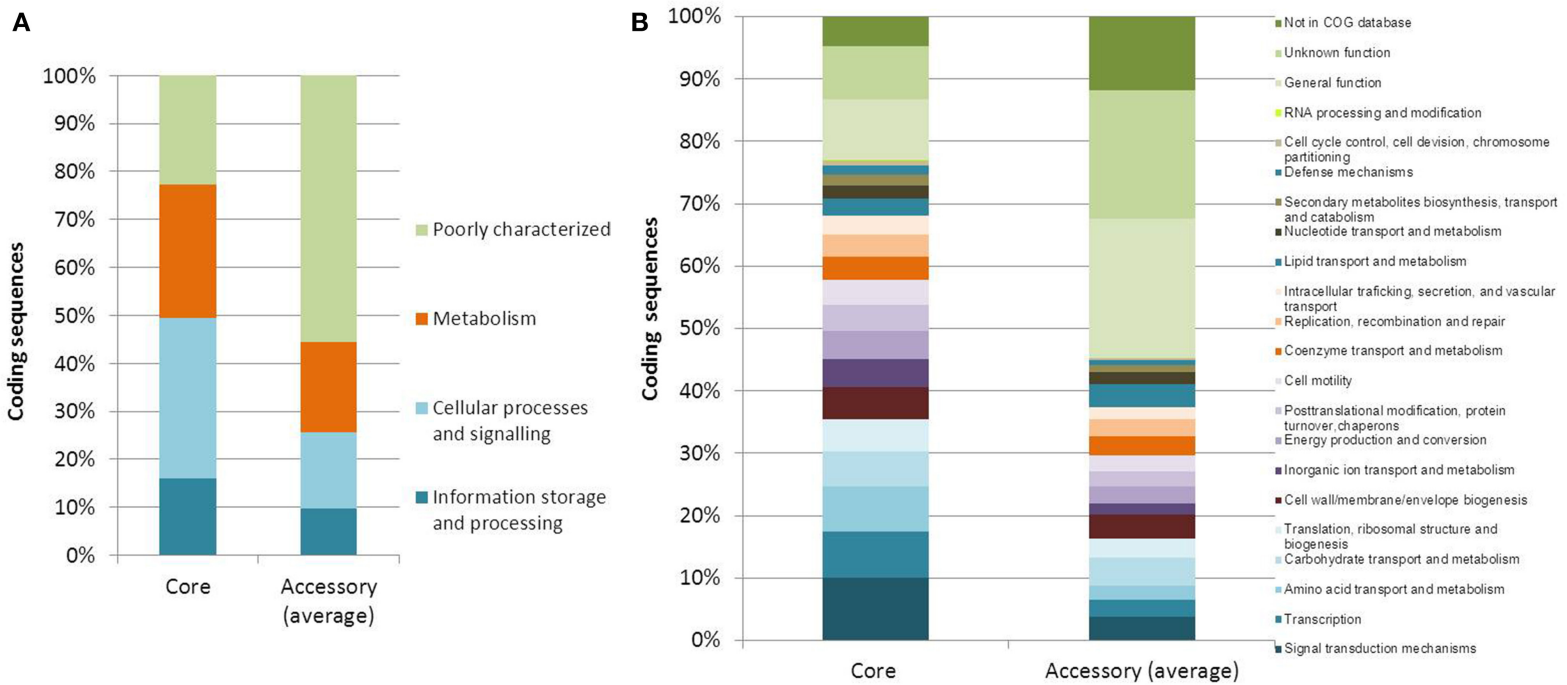
**Functional annotations of core and accessory genes. (A)** COG categories and **(B)** COG subcategories of predicted genes within the core and accessory genomes of *V. vulnificus*. Each category or subcategory is graphed as a percentage of the total number of genes in the core or accessory genomes. Accessory genome percentages are averages of the 16 analyzed genomes.

The core and accessory genomes were studied using Panseq, SPINE, and AGEnt. We included all publicly available genome assemblies in addition to the strains sequenced in the current study and another two biotype 3 strains that were studied by others (VVyb1 and BAA87). We also included the recently published draft genome of biotype 2 strain ATCC 33147. Altogether the analysis included 16 genomes (three complete genomes of the reference strains belonging to the C-genotype and 13 draft genomes). All input sequences from each assembly were concatenated into a single sequence and multiple sequence alignment was produced. The core genome of *V. vulnificus* based on 16 genomes consisted of 3068 genes totaling 3,435,751 bp in size with a GC content of 47.3% (File S2), representing 67% of the whole genome of reference strain CMCP6 (being lowest for the whole genome of biotype 3 strain – VVyb1, 59%, and highest for biotype 1 strain – ATCC 27562, 78%). A total of 214,879 SNPs were identified among the aligned core genomes. The core genome increased as fewer genomes were analyzed reaching 3638 genes when only three genomes representing the three biotypes were used, CMCP6 (biotype 1), ATCC 33147 (biotype 2), and 491771 (biotype 3) (Figure 1). Coding sequences of core and accessory genomes were assigned to functional categories and subcategories using the Clusters of Orthologous Groups of proteins (COG) database (Tatusov et al., 2001) (Figure 2). Nearly 22 and 56% of the core and accessory genomes encoded poorly characterized proteins (including proteins categorized as general function, unknown function, or not in COG database). The average size of the accessory genome of *V. vulnificus* strain was 1662 kbp (being smallest in the clinical strain ATCC 27562, 781 kbp, and largest in the environmental strain 101/4, 2044 kbp). BLAST alignment of the non-core genomes against the reference clinical strain CMCP6 showed that most of the non-core genomic regions aligned to the large chromosome (data not shown). Accessory element composition was diverse, largely consisting of hypothetical proteins, integrative and conjugative elements, prophages and phage-like elements, transposons, insertion sequences, and integrons.

### Characteristic features of biotype 3 genomes

Analysis of sequence variance of the *vcg* gene (virulence correlated gene) (Rosche et al., 2005) showed that all biotype 3 strains were classified as E-genotype. In addition, two environmental biotype 1 strains 101/04 and 2322, biotype 2 strain (ATCC 33147), and two clinical biotype 1 strains, ATCC27562 and B2, were all E-genotype. Clustering and phylogenetic analysis of biotype 3 strains showed that strains VV9-09, VV 4-03, VVyb1, and BAA87 were genetically more closely related and clustered separately from strain 491771. Applying the NRF module of Panseq to search for sequences present in strain 491771 and absent from the other biotype 3 strains resulted in 114,855 bp sequences that resolved into 127 genes, of which the vast majority encoded hypothetical proteins and phage-related proteins (File S3). Biotype 3 strains shared between 85.4 and 89.5% of their genomes with the genomes of the rest of the strains, being highest for the clinical strains CMCP6, YJ016, MO6-24/O, ATCC 27562, and B2 and lowest for the genomes of the environmental strains 2322 and 101/4.

Next we searched for accessory genomic regions found in biotype 3 strains and absent from the core genome of all the strains. Annotation of these accessory genomic sequences yielded 1732 genes, of which the vast majority encoded hypothetical proteins, phage-related proteins, and mobile element proteins (File S4). Several genes encoded plasmid conjugative transfer proteins that displayed high sequence similarity to parts of the genomes of *V. vulnificus* pR99 plasmid, *Vibrio* sp. 04Ya090 plasmid pAQU2, *V. vulnificus* pC4602-1 plasmid, *Vibrio cholerae* plasmid pVC, and *Vibrio* phage kappa proviral DNA. Significant sequence alignment was found to nearly the entire length of *V. cholerae O139* class 4 integron genes for hypothetical protein, *Vibrio parahaemolyticus* HTH gene for HTH-domain protein, *V. vulnificus* super-integron, and insertion sequence ISVpa2 KX-V237 found in many *vibrio* species including *V. parahaemolyticus* O3:K6, *V. vulnificus*, *Vibrio penaeicida*, and *Vibrio splendidus*.

### Phylogenetic analysis

**Figure 3:**
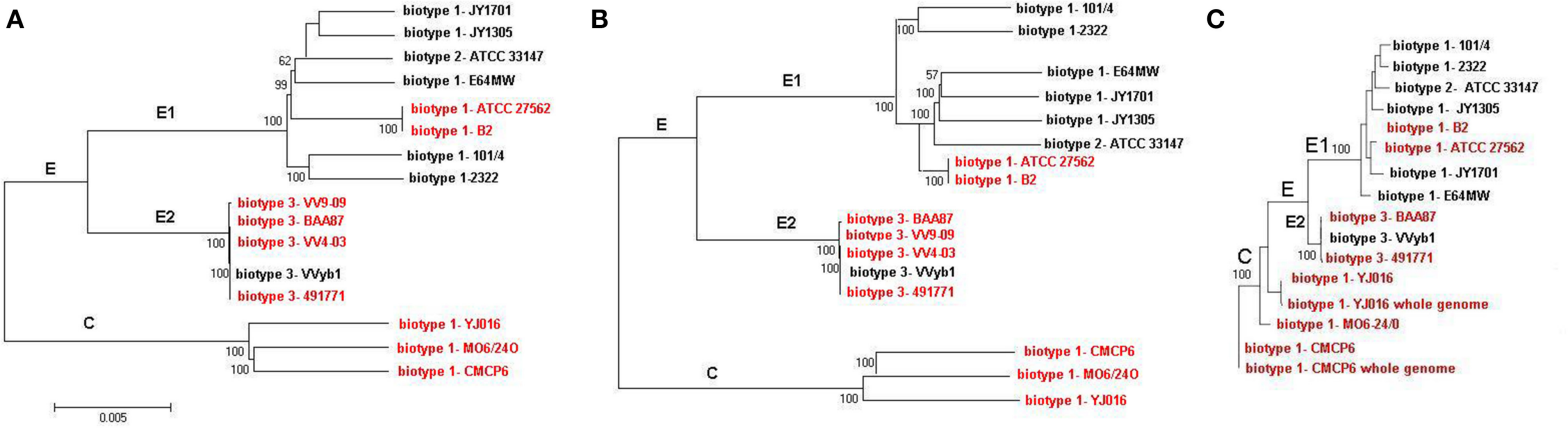
**REALPHY analysis based on WGS sequence data from current study and other publicly available genome sequence data**. **(A)** Based on whole genomes, **(B)** based on core genomes, **(C)** based on accessory genomes. Human pathogenic strains are indicated by red font. Bootstrapping was performed using 500 iterations. For clarity purposes some of the strains were not included in the analysis.

**Figure 4:**
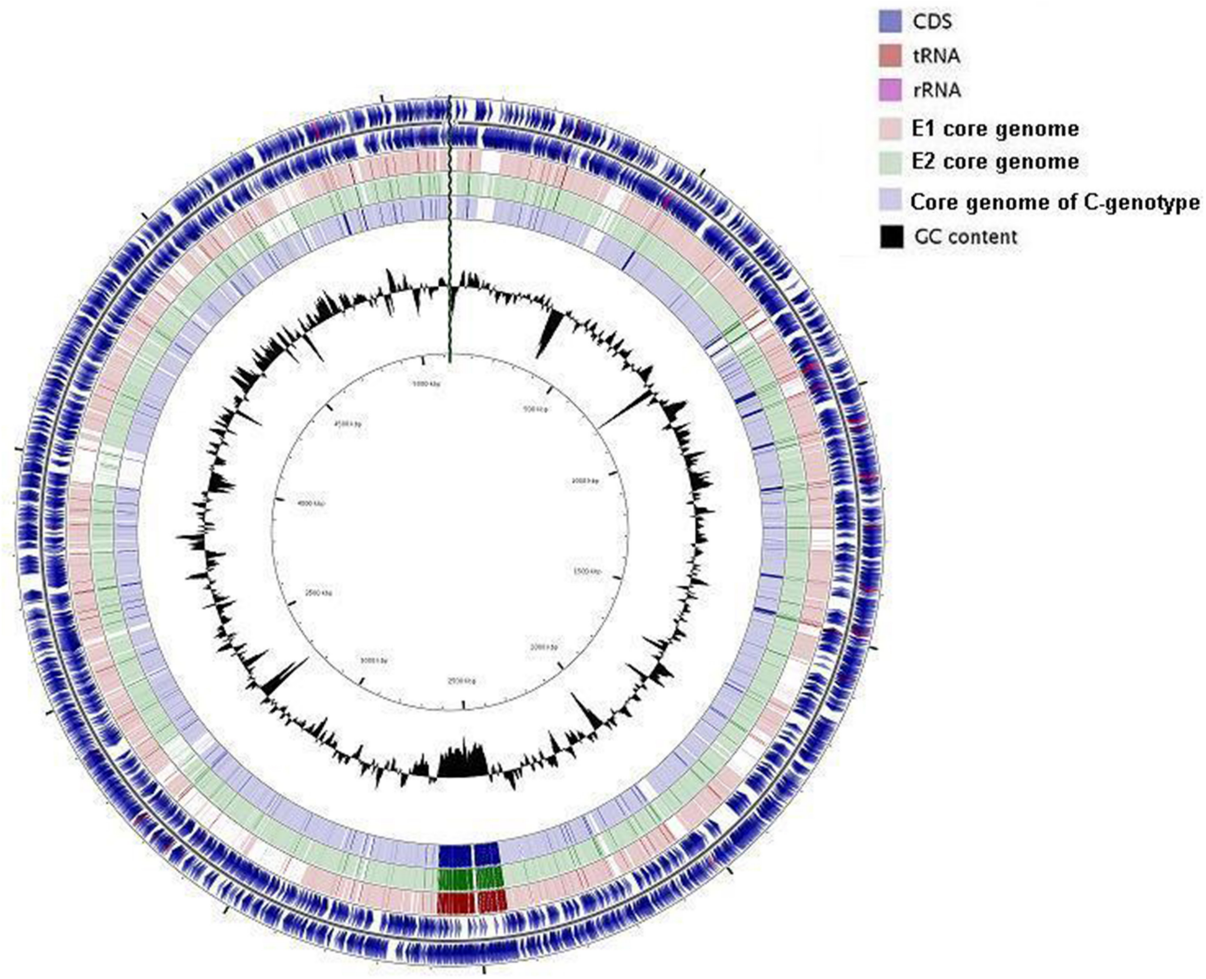
**Circular view of BLAST alignment of three core genomes against the complete genome of reference strain CMCP6**. The circles from outside to inside include; CDS positive and negative strands, group E1 core genome, group E2 core genome, group C core genome, GC content. Figure generated using CGView (Grant and Stothard, 2008).

Phylogenetic analysis was carried out based on whole genomes and then repeated using core and accessory genomes separately. Both analyses showed similar phylogenetic relationships with two distinct clusters consistent with the division into C and E genotype paradigm where the clinical reference genomes clustered separately from the rest of the strains (Figure 3). Two groups were identified within the E cluster, the first, designated E1, included five biotype 1 environmental strains, two biotype 1 clinical strains (ATCC 27562 and B2), and an eel-pathogenic biotype 2 strain (ATCC 33147). All biotype 3 strains clustered separately within the E-cluster and formed another group designated E2 (Figure 3A). Repeating the analysis using core genomes (Figure 3B) and accessory genomes (Figure 3C) resulted in similar clustering pattern with minor differences in grouping the strains within the clusters. BLAST alignment of the core genomes of groups E1, E2, and cluster C against the complete genome sequence of clinical reference strain CMCP6 showed that the core genome of biotype 3 strains (E2) was the largest and shared more regions with the core genome of cluster C than with group E1(Figure 4). The genomic regions found in group E2 and absent from group E1 consisted of 259 contigs totaling 1,407,644 bp in size, from which 1273 genes were identified. The largest group consisted of genes encoding hypothetical proteins (32%), while other groups were made up of phage-related proteins, mobile elements proteins, genes involved in DNA metabolism, and genes encoding membrane transport proteins.

In order to characterize the set of sequences shared by the human-pathogenic strains and absent from environmental strains we compared each and every human-pathogenic strain (VV9-09, VV 4-03, 491771 from the present study, complete genomes of three reference human pathogenic strains; CMCP6, YJ016, MO6-24/O, and WGS sequence data from strains BAA87, ATCC275962, and B2) against all five environmental strains (101/04, 2322, E64MW, JY1701, and JY1305 – E1 group in Figure 3). A total of 1916 sequences/proteins (including 713 hypothetical proteins) were present in the human pathogenic strains and absent from the environmental strains. Clustering analysis of the non-hypothetical proteins (*n* = 1203) revealed 148 protein clusters shared by the human-pathogenic strains (File S5). The proteins shared among the pathogenic strains included transcriptional regulators, arylsulfatases, methyl-accepting chemotaxis proteins, acetyltransferases, GGDEF family proteins, transposases, type IV secretory system (T4SS) proteins, and integrases. The 713 hypothetical proteins resolved into 319 clusters consisting largely of proteins shared by the same phylogenetic group: that is clusters unique to the reference genomes, clusters unique to biotype 3 strains, and clusters unique to strains ATCC 27562 and B2 present within E-genotype population, probably indicative of their common ancestral origin. Very few hypothetical protein clusters were shared by all the human pathogenic strains.

## Discussion

We used sequence data from 16 genomes (draft and complete) to study the pan genome of *V. vulnificus* and investigate the evolutionary relationship of biotype 3 with existing populations. The core genome of *V. vulnificus* consisted of 3068 genes comprising nearly two-thirds of the set of genes of the complete genome sequence of three reference genomes. All biotype 3 strains resolved into the E-genotype cluster yet formed a distinct group from the rest of the strains. The core genome of biotype 3 strains (group E2) was the largest among the dataset, consistent with its recent evolution and highly clonal nature as previously described (Bisharat et al., 2007a), while the core genome of group E1 was the smallest consistent with the genetic diversity of environmental populations. Annotation of the genomic regions found in in the core genome of biotype 3 strains and absent from the core genome of group E1 strains showed that nearly a third of the genes encoded hypothetical proteins in addition to many phage related and mobile element proteins. The core genome of biotype 3 strains (E2) shared more regions with the core genome of the clinical reference strains (cluster C) than with the core genome of the other environmental group (E1) (Figure 4). This is likely due to the relatively small number of strains included in the analysis (three reference clinical strains representing cluster C and five strains representing group E2, while group E1 was represented by eight strains). Using an equal and rather large number of strains from each group, would have probably resulted in minor changes in the size of the core genome of each group and largely representing the core genome of the species.

Phylogenetic analysis divided the dataset into two distinct populations, in agreement with previous studies (Gutacker et al., 2003; Rosche et al., 2005; Bisharat et al., 2007b). This structuring pattern of *V. vulnificus* populations into two distinct clusters has been observed across housekeeping genes (Bisharat et al., 2005), genes encoding outer membrane proteins (Bisharat et al., 2007a), 16s rRNA genes (Aznar et al., 1994), and across whole genomes (current study). In addition, these observations were confirmed in various geographical regions, USA (Rosche et al., 2005; Warner and Oliver, 2008; Reynaud et al., 2013), Baltic Sea region (Bier et al., 2013), South and Southeast Asia (Mahmud et al., 2008, 2010), and the Middle East (Bisharat et al., 2007b). Furthermore, inferred phylogeny from current study based on whole, core, or accessory genomes resulted in nearly identical clustering pattern. Overall these findings strongly imply that *V. vulnificus* populations may have diverged into two main clusters in ancient times. Nevertheless, the emergence of a new genotype with unique phenotypic profile in Israel, suggested that a new genetically distinct population may have existed and affected public health due to human behavior (Bisharat and Raz, 1996). Subsequent molecular analysis suggested that this genotype may have emerged due to recombination between the two distinct populations (Bisharat et al., 2005). Data from current study suggests that biotype 3 is a clone that diverged from the parental population, cluster E. This clonal lineage may have emerged and succeeded due to the acquisition of a strong selective advantage, allowing it to rise in frequency in the population and eventually creating a distinct lineage (Smith et al., 1993). The genetic distinction of group E2 (biotype 3) from the parent population and from the C-genotype cluster was not driven by the accessory genome as core-genome and accessory-genome based analysis exhibited similar phylogenetic relationships. We have previously emphasized the role of recombination and mutation in generating genetic diversity within *V. vulnificus* and suggested that it may have impacted the evolution of biotype 3 strains (Bisharat et al., 2007b). Nevertheless, it seems that the accessory genome has likely evolved together with the core genome in view of the fixed clustering pattern observed over different genomic levels of analysis.

A yet unanswered question is why human disease in Israel is almost entirely caused by biotype 3 (Bisharat et al., 2005; Zaidenstein et al., 2008). Previous studies by our group and others have identified strains, exhibiting non-biotype 3 genotype, belonging to both clusters in the environment (Bisharat et al., 2007b; Broza et al., 2009). Is it because biotype 3 has better fitness or greater virulent potential than other genotypes, or is it because of higher frequencies within the water or fish? Previous environmental surveys conducted in Israel in fish farms during 2004–2006 have shown that biotype 3 frequencies ranged from 2 to 21% of environmental populations which largely consisted of biotype 1 strains (Broza et al., 2009). We speculate that biotype 3 genotype is a subtype of the E-cluster that is of greater virulent potential than other E or C genotypes circulating in fish farms. Luckily, *V. vulnificus* disease burden in Israel have decreased dramatically in the past 10 years and it's restricted now to fish farm associated activities (farming, fishing, and maintenance). In addition, the population at risk has decreased dramatically due to preventive measures aimed to increase the awareness of the population to this dreadful pathogen and decrease risk of infection by changing fish marketing policies (Bisharat, 2002).

The list of protein clusters shared by the human pathogenic strains and absent from the environmental strains is similar to reports published by others in recent years (Gulig et al., 2010; Morrison et al., 2012). Nearly all the genes/proteins shared by the human pathogenic biotype 3 strains were found in all the strains tested, while not all the listed genes were found in all the clinical reference genomes, suggesting that some genes are not entirely involved in virulence and may have other functional roles.

The epidemiology of *V. vulnificus* disease in Israel was unique and unexpected, and the only common source outbreak reported to date. It erupted due to changes in fish marketing policies where tilapia fish were sold live in freshwater instead of dead and packed in ice (Bisharat and Raz, 1996). Had this not occurred, the emergence of biotype 3 may have gone undetected, causing only sporadic cases as occurred in some European countries (Melhus et al., 1995; Dalsgaard et al., 1996; Garcia Cuevas et al., 1998; Horre et al., 1998; Torres et al., 2002; Mitra, 2004; Frank et al., 2006).

Our study showed that *V. vulnificus* biotype 3 is a distinct clone that have descended from the parental environmental population and may have acquired pathogenic potential by lateral gene transfer from other *vibrios* thus enabling a harmless environmental species to cause disease in humans. The unique epidemiological circumstances facilitated disease outbreak and brought this genotype to the attention of the scientific community. These novel observations reveal yet another way by which epidemic organisms arise.

## Author contributions

Yael Koton wrote the initial draft, Michal Gordon and Vered Chalifa-Caspi carried out part of the bioinformatics analysis. Naiel Bisharat planned, coordinated, analyzed data, and wrote the manuscript.

### Conflict of interest statement

The authors declare that the research was conducted in the absence of any commercial or financial relationships that could be construed as a potential conflict of interest.
